# Tolvaptan and Kidney Function Decline in Older Individuals With Autosomal Dominant Polycystic Kidney Disease: A Pooled Analysis of Randomized Clinical Trials and Observational Studies

**DOI:** 10.1016/j.xkme.2023.100639

**Published:** 2023-04-14

**Authors:** Fouad T. Chebib, Xiaolei Zhou, Diana Garbinsky, Eric Davenport, Sasikiran Nunna, Dorothee Oberdhan, Ancilla Fernandes

**Affiliations:** 1 Division of Nephrology and Hypertension, Mayo Clinic, Jacksonville, FL; 2 RTI Health Solutions, Research Triangle Park, NC; 3 Otsuka Pharmaceutical Development & Commercialization, Inc, Rockville, MD

**Keywords:** Autosomal dominant polycystic kidney disease (ADPKD), clinical trial, glomerular filtration rate (GFR), subgroup analysis, tolvaptan

## Abstract

**Rationale & Objective:**

Tolvaptan is indicated for treatment of patients with autosomal dominant polycystic kidney disease (ADPKD) at risk of rapid progression. Participants aged 56-65 years constituted a small proportion of the Replicating Evidence of Preserved Renal Function: an Investigation of Tolvaptan Safety and Efficacy in ADPKD (REPRISE) trial population. We assessed effects of tolvaptan on estimated glomerular filtration rate (eGFR) decline in participants aged >55 years.

**Study Design:**

This was a pooled data analysis from 8 studies of tolvaptan or non-tolvaptan standard of care (SOC).

**Setting & Participants:**

Participants aged >55 years with ADPKD were included. Data on participants in >1 study were linked longitudinally for maximum follow-up duration, with matching for age, sex, eGFR, and chronic kidney disease (CKD) stage to minimize confounding.

**Interventions:**

Tolvaptan or non-tolvaptan SOC.

**Outcomes:**

Treatment effects on annualized eGFR decline were compared using mixed models with fixed effects for treatment, time, treatment-by-time interaction, and baseline eGFR.

**Results:**

In the pooled studies, 230 tolvaptan-treated and 907 SOC participants were aged >55 years at baseline. Ninety-five participant pairs from each treatment group were matched, all in CKD G3 or G4, ranging from 56.0 to 65.0 years (tolvaptan) or from 55.1 to 67.0 years (SOC). The eGFR annual decline rate was significantly reduced by 1.66 mL/min/1.73 m^2^ (95% CI, 0.43-2.90; *P* = 0.009) in the tolvaptan group compared with SOC (−2.33 versus −3.99 mL/min/1.73 m^2^) over 3 years.

**Limitations:**

Limitations include potential bias because of study population differences (bias risk was reduced through matching and multiple regression adjustment); vascular disease history data was not uniformly collected, and therefore not adjusted; and natural history of ADPKD precludes evaluating certain clinical endpoints within the study time frame.

**Conclusions:**

In individuals aged 56-65 years with CKD G3 or G4, compared to a SOC group with mean GFR rate of decline ≥3 mL/min/1.73 m^2^/year, tolvaptan was associated with efficacy similar to that observed in the overall indication.

**Funding:**

Otsuka Pharmaceutical Development & Commercialization, Inc (Rockville, MD).

**Trial Registration:**

TEMPO 2:4 (NCT00413777); phase 1 tolvaptan trial (no NCT number; trial number 156-06-260); phase 2 tolvaptan trial (NCT01336972); TEMPO 4:4 (NCT01214421); REPRISE (NCT02160145); long-term tolvaptan safety extension trial (NCT02251275); OVERTURE (NCT01430494); HALT Progression of Polycystic Kidney Disease (HALT-PKD) study B (NCT01885559).

## Graphical abstract

**Figure:**
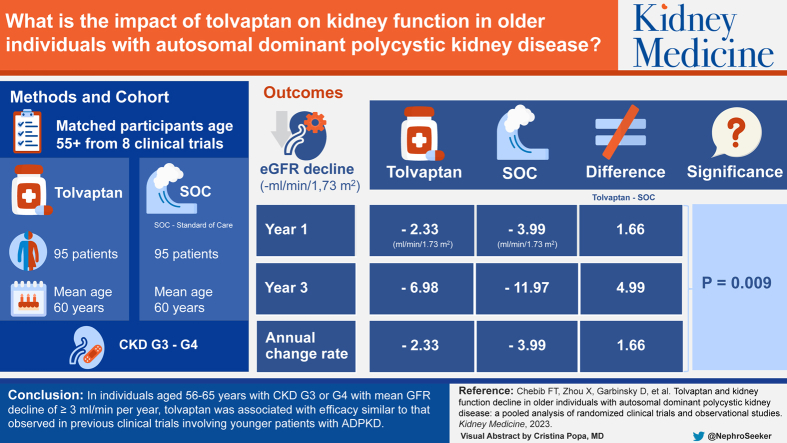

**Plain-Language Summary**

Data are lacking on tolvaptan efficacy in slowing kidney function decline among patients with autosomal dominant polycystic kidney disease aged >55 years. The REPRISE trial enrolled patients in this group, but the numbers were small, and follow-up was only 1 year. We pooled data from multiple studies of tolvaptan or non-tolvaptan standard of care to explore efficacy of tolvaptan in older patients. Data for individuals who participated in multiple studies were linked longitudinally to enable longer follow-up, with patient matching for baseline age, kidney function, and sex. Among 95 matched patient pairs (ages 55-67 years, chronic kidney disease stage G3 or G4), statistical modeling indicated significant slowing of annualized eGFR decline with tolvaptan versus standard of care over 3 years of follow-up (−2.33 versus −3.99 mL/min/1.73 m^2^/year; *P* = 0.009).

Tolvaptan is the only disease-specific treatment for autosomal dominant polycystic kidney disease (ADPKD) and is indicated to slow kidney function decline in patients who are at high risk of rapid progression.^1^ The TEMPO 3:4 clinical trial (NCT00428948) demonstrated that tolvaptan slowed kidney function decline and total kidney volume expansion over 3 years relative to placebo in a large population (N=1445) of high-risk patients.^2^ Eligibility criteria were age 18-50 years with largely preserved kidney function (estimated creatinine clearance ≥60 mL/minute) and large total kidney volume (>750 mL). At baseline, the study population had a mean age of 39 years and chronic kidney disease (CKD) stage distribution of 35% G1, 48% G2, and 17% G3.^2^^,^^3^

In the Replicating Evidence of Preserved Renal Function: an Investigation of Tolvaptan Safety and Efficacy in ADPKD (REPRISE) trial (NCT02160145) conducted in patients with more advanced ADPKD, tolvaptan exhibited significant reduction versus placebo in the rate of estimated glomerular filtration rate (eGFR) decline in 1,331 participants over 1 year. By entry criteria, the population in REPRISE was older with more advanced CKD than in TEMPO 3:4; eligible patients were either aged 18-55 years with eGFR 25-65 mL/min/1.73 m^2^ or 56-65 years with eGFR 25-44 mL/min/1.73 m^2^ and historical evidence of rapid eGFR decline.^4^ Mean age at baseline was 47 years, and the CKD distribution was 5% G2, 75% G3, and 20% G4. All enrolled participants initially received tolvaptan in a 2-week titration period followed by a 3-week tolvaptan run-in period. Participants who completed the run-in period and tolerated tolvaptan 60/30 mg/day or 90/30 mg/day were randomized to receive either tolvaptan or placebo in the 12-month double-blind treatment period.

Although patients aged >55 years were eligible to enroll in REPRISE, the actual number of such participants was limited, with 96 in the tolvaptan arm and 94 in the placebo arm who could be evaluated for the primary efficacy endpoint. Furthermore, patients aged >55 years who received placebo had slower than anticipated disease progression, as evidenced by their annualized eGFR decline (−2.34 mL/min/1.73 m^2^) compared with those in the placebo arm aged ≤55 years (−4.60 mL/min/1.73 m^2^). Rates of eGFR decline for tolvaptan and placebo in the >55 year old subgroup were therefore nearly indistinguishable. Recommendations on the use of tolvaptan in ADPKD have accordingly focused on benefits for patients aged 18-55 years.^5^

Since the pivotal clinical trials of tolvaptan were conducted, additional follow-up data on tolvaptan-treated patients from extension trials have become available.^6^^,^^7^ Conducting additional long-term, placebo-controlled trials of a treatment that has demonstrated efficacy may not be feasible and is undesirable from an ethical perspective. Given this situation, data from long-term clinical or observational studies of patients with ADPKD who were not treated with tolvaptan can be used as a control cohort to investigate long-term outcomes. Such data have recently become available from the OVERTURE study (NCT01430494).^8^

Understanding potential benefits of tolvaptan use in patients with ADPKD aged >55 years and at later stages of CKD could help patients and their health care providers make informed treatment decisions. To further characterize the effects of tolvaptan on the trajectory of kidney function decline over the long term, we analyzed a large, pooled database from tolvaptan clinical trials and non-tolvaptan studies on this patient subset.

## Methods

### Design

This pooled database analysis compared the trajectory of kidney function over time in populations enrolled in tolvaptan clinical trials or natural history studies in which standard of care (SOC) management that did not include tolvaptan was provided. The dataset and analytical methods have been previously described in detail.^9^

The present analysis included individuals aged >55 years at baseline. Data for this patient subgroup were available from clinical studies that were sponsored by Otsuka or the National Institutes of Health and conducted from 2005 to 2018 (Table S1): REPRISE, TEMPO 2:4 (NCT00413777),^10^ a phase 1 tolvaptan trial (no NCT number; trial number 156-06-260),^11^ a phase 2 tolvaptan trial (NCT01336972),^12^ TEMPO 4:4 (NCT01214421),^6^ a long-term tolvaptan safety extension trial (NCT02251275),^7^ OVERTURE,^8^ and HALT Progression of Polycystic Kidney Disease (HALT-PKD) study B (NCT01885559).^13^ In the observational OVERTURE study, SOC was defined as the treatment approach selected by the participant’s individual physician; this study was conducted before the commercial availability of tolvaptan for ADPKD.^8^ In HALT-PKD study B, the study treatments were various antihypertensive regimens for blood pressure control 110-130/70-80 mm Hg.^13^ As participants may have entered subsequent studies after their participation in an initial study concluded, data were linked longitudinally across studies to enable long-term follow-up of unique participants.

Calculation of eGFR was performed using the Chronic Kidney Disease Epidemiology Collaboration (CKD-EPI) equations.^14^ Serum creatinine was measured by the enzymatic method in all studies, except for the long-term extension trial of tolvaptan in which serum creatinine was measured by the rate blank method.^15^ In REPRISE, serum creatinine was analyzed by both the enzymatic and rate blank methods. Although eGFR data obtained by each method show the same change over time, they are not interchangeable at a single assessment. When assessing change in eGFR, the same measurement method for serum creatinine should be used for unique individuals who participated in multiple studies. Therefore, in the present analyses, eGFR based on rate blank serum creatinine measurement was used for postbaseline values for REPRISE and the subsequent long-term extension. For individuals who originally participated in studies other than REPRISE, only enzymatic serum creatinine measurements collected in those studies were used, and the rate blank-derived values collected in the long-term extension were not included in the analyses. For all participants, eGFR derived from enzymatic serum creatinine measurement was used for the baseline value. Differences in the timing of eGFR assessment in the clinical studies (Table S2) were accounted for by the statistical modeling described below.

### Statistical Analyses

Differences in baseline characteristics between the tolvaptan and SOC groups were analyzed using standardized mean difference, with values of 0.2 or greater indicative of between-group differences.^16^ The outcome of interest in this study was annual change in eGFR (mL/min/1.73 m^2^ per year). To minimize confounding effects, tolvaptan-treated participants were matched 1:1 with SOC participants for baseline CKD stage, sex, age (±2 years), and eGFR (±5 mL/min/1.73 m^2^) to derive a matched analysis set. Matching was conducted using the %gmatch SAS macro developed by the Mayo Clinic based on the greedy method. The matching ratio was 1:1. By study design, all enrolled participants in REPRISE received tolvaptan during a 5-week tolvaptan titration and run-in period before randomization to tolvaptan or placebo, and only participants randomized to tolvaptan were eligible for matching. The matched treatment groups were compared using mixed models with fixed effects for treatment, time (as a continuous variable), treatment-by-time interaction, and baseline eGFR.

For the overall group of participants aged >55 years identified from the database, piecewise mixed models were applied as reported to assess the treatment effects of tolvaptan.^9^ This method was selected to account for gaps in tolvaptan treatment by using different slope parameters for time in the tolvaptan treatment period and time in the tolvaptan gap period. The models included participant-specific intercept and slope (for time) as random effects with an unstructured variance-covariance matrix. Estimates from the piecewise mixed models were adjusted for baseline eGFR, age, sex, race (White vs other), height, systolic blood pressure, diastolic blood pressure, history of hematuria, history of urinary tract infection, history of nephrolithiasis, and time in the tolvaptan gap period.

Off-treatment observations in the tolvaptan cohort were excluded from modeling because of the hemodynamic effect of tolvaptan (an acute reduction in eGFR right after treatment initiation, which is reversible once patients are off treatment).^11^^,^^12^ Specifically, for participants in the tolvaptan cohort, eGFR assessments <7 days after tolvaptan initiation, during a tolvaptan treatment gap, or after tolvaptan treatment termination were excluded. For the same reason of excluding the hemodynamic effect, changes from “baseline” eGFR were estimated based on the theoretical baseline value estimated from the mixed models.

## Results

### Analysis Populations

**Figure 1:**
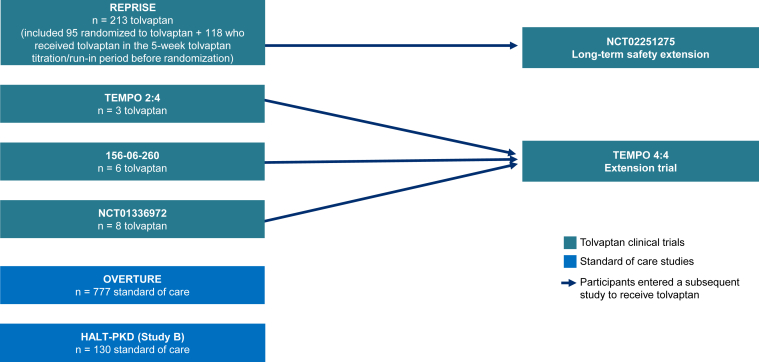
Source studies for the entire pooled dataset of participants aged >55 years.

In the entire pooled dataset, 230 patients from the tolvaptan group and 907 patients from the SOC group were aged >55 years (Fig 1). Most of the patients in the tolvaptan group were from REPRISE (n=213), and the remaining 17 were from other studies (3 from TEMPO 2:4, 6 from phase 1 trial 156-06-260, and 8 from phase 2 trial NCT01336972). Patients in the SOC group were from OVERTURE (n=777) and HALT-PKD study B (n=130).

Baseline demographics and clinical characteristics were generally similar between the tolvaptan and SOC groups (Table 1), but the tolvaptan group had higher proportions of participants in CKD stages G3b and G4 and with histories of nephrolithiasis, hematuria, and urinary tract infection. For many participants, data was missing for age at onset of hypertension, which was not available in phase 1 trial 156-06-260 and OVERTURE, and for baseline total kidney volume and related variables, which were not available for REPRISE and HALT-PKD study B.

**Table 1:** Baseline Characteristics of All Participants Aged >55 Years and of the Matched Analysis Set

Characteristic	All Aged >55 y			Matched Participants		
	Tolvaptan (n=230)	Standard of Care (n=907)	Standardized Mean Difference	Tolvaptan (n=95)	Standard of Care (n=95)	Standardized Mean Difference
Age (y), n	230	907	—	95	95	—
Mean (SD)	59.8 (2.8)	61.2 (4.3)	−0.37	60.2 (2.9)	60.2 (2.9)	−0.01
Age range, y	55.2-70.0	55.0-78.2	—	56.0-65.0	55.1-67.0	—
Female, n (%)	129 (56.1%)	476 (52.5%)	0.07	51 (53.7%)	51 (53.7%)	0.00
Race, n (%)	230	907	—	95	95	—
White	207 (90.0%)	760 (83.8%)	0.18	89 (93.7%)	87 (91.6%)	0.08
Black	8 (3.5%)	22 (2.4%)	0.06	2 (2.1%)	0	0.21
Hispanic	8 (3.5%)	107 (11.8%)	−0.32	3 (3.2%)	5 (5.3%)	−0.10
Asian	2 (0.9%)	10 (1.1%)	−0.02	0	2 (2.1%)	−0.21
Other	5 (2.2%)	8 (0.9%)	0.11	1 (1.1%)	1 (1.1%)	0.00
Body mass index in kg/m^2^, n	228	893	—	94	95	—
Mean (SD)	27.8 (5.2)	27.7 (4.8)	0.03	27.4 (5.0)	27.9 (5.0)	−0.09
Age at ADPKD diagnosis (y), n	230	903	—	95	95	—
Mean (SD)	40.0 (12.3)	45.4 (12.5)	−0.43	40.2 (12.6)	43.5 (11.8)	−0.28
Chronic kidney disease stage, mL/min/1.73 m^2^, n	210	879	—	95	95	—
≥90 (G1), n (%)	0	39 (4.4%)	−0.30	0	0	—
60 to <90 (G2), n (%)	5 (2.4%)	205 (23.3%)	−0.66	0	0	—
45 to <60 (G3a), n (%)	2 (1.0%)	202 (23.0%)	−0.72	66 (69.5%)^a^	66 (69.5%)^a^	−0.00
30 to <45 (G3b), n (%)	146 (69.5%)	231 (26.3%)	0.96			
15 to <30 (G4), n (%)	57 (27.1%)	144 (16.4%)	0.26	29 (30.5%)	29 (30.5%)	−0.00
<15 (G5), n (%)	0	58 (6.6%)	−0.38	0	0	—
Baseline eGFR in mL/min/1.73 m^2^, n	210	879	—	95	95	—
Mean (SD)	34.2 (8.1)	47.2 (22.1)	−0.78	33.3 (5.3)	33.7 (5.8)	−0.08
eGFR range, mL/min/1.73 m^2^	16.6-84.2	3.4-106.5	—	22.5-43.9	22.0-46.2	—
Baseline systolic BP in mm Hg, n	230	900	—	95	95	—
Mean (SD)	132.0 (16.0)	134.3 (17.5)	−0.13	131.3 (16.4)	131.8 (17.1)	-0.03
Baseline diastolic BP in mm Hg, n	230	900	—	95	95	—
Mean (SD)	80.9 (10.0)	80.2 (10.8)	0.06	79.2 (10.0)	78.2 (10.3)	0.10
Age at hypertension onset (y)^b^, n	209	130	—	88	53	—
Mean (SD)	43.5 (9.9)	43.0 (10.7)	0.05	43.4 (10.0)	41.9 (12.5)	0.14
ADPKD-related complications reported^b^, n	224	907	—	95	95	—
History of nephrolithiasis, n (%)	45 (20.1%)	65 (7.2%)	0.38	23 (24.2%)	6 (6.3%)	0.51
History of hematuria, n (%)	50 (22.3%)	119 (13.1%)	0.24	21 (22.1%)	9 (9.5%)	0.35
History of urinary tract infection, n (%)	61 (27.2%)	80 (8.8%)	0.49	32 (33.7%)	7 (7.4%)	0.69
ADPKD risk classification^b^, n	17	716	—	—	—	—
Class 1A, n (%)	2 (11.8%)	81 (11.3%)	0.01	—	—	—
Class 1B, n (%)	4 (23.5%)	262 (36.6%)	−0.29	—	—	—
Class 1C, n (%)	9 (52.9%)	299 (41.8%)	0.23	—	—	—
Class 1D, n (%)	0	69 (9.6%)	−0.46	—	—	—
Class 1E, n (%)	2 (11.8%)	5 (0.7%)	0.47	—	—	—

Abbreviations: ADPKD, autosomal dominant polycystic kidney disease; BP, blood pressure; eGFR, estimated glomerular filtration rate; SD, standard deviation.

a 30 to <60 mL/min/1.73 m^2^ (stage G3). All 66 participants in the tolvaptan group and 63 of 66 participants in the standard of care group were in stage G3b (30 to <45 mL/min/1.73 m^2^).

b Not assessed in all studies.

Application of the matching criteria yielded an analysis set of 95 participant pairs, which included all participants aged >55 years at baseline who were randomized to tolvaptan in REPRISE. Of the 213 tolvaptan-treated participants over age 55 in REPRISE who were included in the overall pooled dataset, 118 were excluded from matching because they had received tolvaptan for only 5 weeks in the prerandomization titration/run-in period of REPRISE and were not randomized to the tolvaptan arm. These individuals were later allowed to resume tolvaptan in the long-term safety extension study. Thus, 95 participants from the tolvaptan treatment arm were eligible for matching (Table S3). As intended by the matching procedure, the tolvaptan and SOC groups were well aligned in terms of age, sex, and kidney function (Table 1). The tolvaptan and SOC groups each had a mean age of 60.2 years (range of 56.0-65.0 for tolvaptan and 55.1-67.0 for SOC), 53.7% were female, 69.5% were in CKD G3, and 30.5% were in CKD G4.

### Duration of Follow-up and Tolvaptan Exposure

In the overall pooled analysis set of participants aged >55 years, the mean (standard deviation) duration of follow-up was 2.4 (1.4) years for tolvaptan and 2.2 (1.6) years for SOC. Fifty-nine patients in the tolvaptan group and 112 patients in the SOC group had at least 3 years of follow-up.

**Figure 2:**
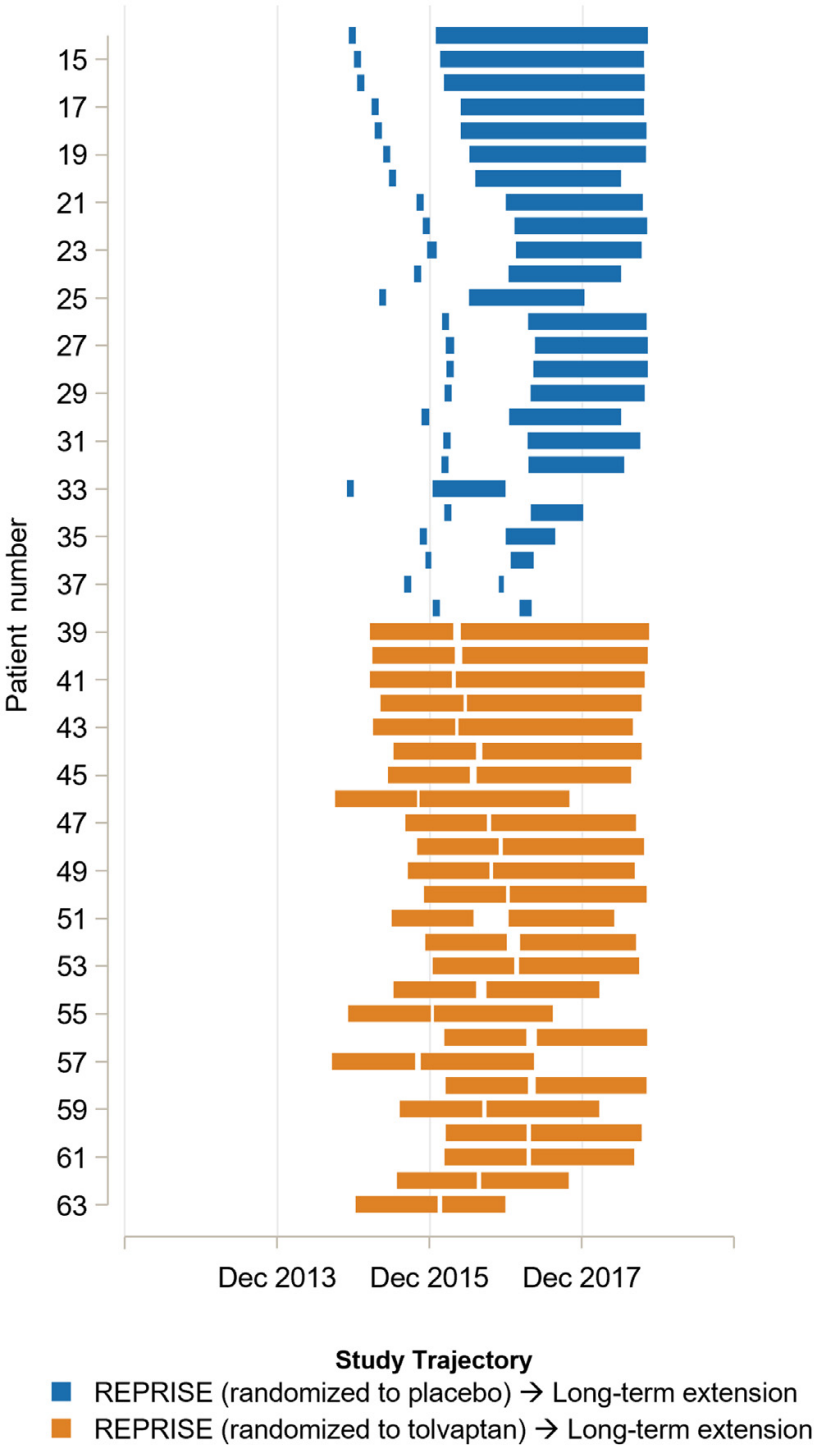
Tolvaptan treatment and treatment gaps in a random sample of participants rolling over from REPRISE to the long-term extension study.

For tolvaptan-treated participants, the mean (standard deviation) duration of tolvaptan treatment was 2.3 (1.5) years, and the mean time actually taking tolvaptan during the treatment period was 1.8 (1.3) years (Table S3). The mean “compliance” rate (percentage of days taking tolvaptan during treatment) was 81.7%. Off-tolvaptan days during treatment were largely because of gaps between tolvaptan use in an initial trial and a subsequent extension trial among participants who participated in more than 1 trial. This gap was longest for participants not randomized to the tolvaptan arm in REPRISE; following an initial tolvaptan titration and run-in period at the start of REPRISE, those from this group who entered the randomized phase of the study received placebo before the long-term extension, yielding a mean (standard deviation) gap in tolvaptan use of 1.1 (0.1) years (Table S3). Time on tolvaptan is shown for a random sample of participants who rolled over from REPRISE into the long-term extension in Fig 2.

### Effects of Treatment on eGFR Decline

In the overall population aged >55 years, the estimated annual rate of eGFR decline was slower with tolvaptan (−2.45 mL/min/1.73 m^2^; 95% confidence interval [CI], −3.48 to −1.42) than with SOC (−3.26 mL/min/1.73 m^2^; 95% CI, −3.84 to −2.68), but the difference did not reach significance (0.81 mL/min/1.73 m^2^; 95% CI, −0.37 to 1.99; *P* = 0.178) in the unmatched population. A subgroup analysis of the overall population aged >55 years and in CKD stages G3 or G4 using the piecewise mixed model yielded an annual rate of eGFR decline of −2.40 mL/min/1.73 m^2^ for tolvaptan and −3.33 mL/min/1.73 m^2^ for SOC, with a significant difference of 0.93 mL/min/1.73 m^2^ (95% CI, 0.28-1.58; *P* = 0.005).

**Figure 3:**
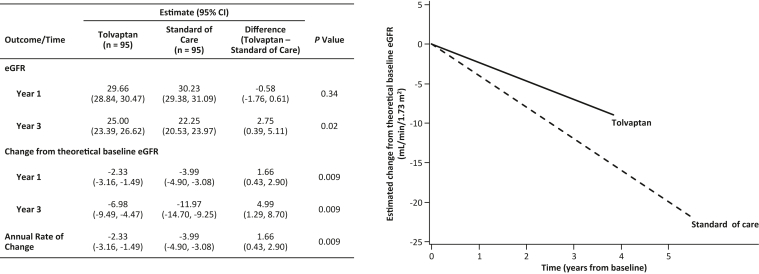
Decline in eGFR over time by treatment cohort in the matched population. eGFR was estimated based on the baseline sample mean of eGFR. Change from “baseline” eGFR was estimated based on the theoretical baseline value estimated from the mixed model that accounted for the hemodynamic effect. CI, confidence interval; eGFR, estimated glomerular filtration rate.

In the matched population, the mixed model indicated that over 3 years of follow-up, the annual eGFR decline rate was −2.33 mL/min/1.73 m^2^ (95% CI, −3.16 to −1.49) with tolvaptan and −3.99 mL/min/1.73 m^2^ (95% CI, −4.90 to −3.08) with SOC, which represented a significant reduction in the annual rate of decline with tolvaptan by 1.66 mL/min/1.73 m^2^ (95% CI, 0.43-2.90; *P* = 0.009) (Fig 3). Change in eGFR over time for each participant in the matched analysis set is shown in Fig S1.

## Discussion

This comparison of long-term data on tolvaptan-treated patients and controls with ADPKD supports an association of tolvaptan with significant improvement in the rate of kidney function decline among patients aged 56-65 years with low eGFR (G3 or G4) and rapid progression evidenced by a historical GFR rate of decline of ≥3 mL/min/1.73 m^2^/year. The beneficial effect of tolvaptan in the treatment of ADPKD was not clear for patients older than 55 years in REPRISE, as participants aged >55 who received placebo had unexpectedly slow progression.^4^^,^^5^ Although randomized clinical trials are the gold standard for evaluating treatment efficacy, subgroup analyses of clinical trial data dilute statistical power, necessitating other analytical methods such as patient matching.^17^ The present pooled data study enabled analysis of a sample that yielded a well-matched set of participant pairs who were treated with either tolvaptan or SOC for a period longer than the REPRISE study. Based on the present study, tolvaptan treatment was associated with a slowing of the annual rate of GFR decline by 1.66 mL/min/1.73 m^2^ per year in older patients with ADPKD (average age 60 years, range 56 to 65) with evidence of rapid progression. In this study, the SOC group had evidence of rapid progression, with an annual eGFR decline rate of −3.99 mL/min/1.73 m^2^ (95% CI, −4.90 to −3.08). In clinical practice and a recent guideline, rapid progression is defined as a historical decline ≥3 mL/min/1.73 m^2^ per year.^18^^,^^19^

Although patients with more advanced CKD have less kidney function to preserve than patients who begin therapy at an earlier stage of their disease, the results of our analysis nonetheless suggest that tolvaptan treatment was associated with a significant benefit in older patients with advanced disease. The matched population selected for this analysis ranged in age from 55 to 67 years across the tolvaptan and SOC groups and were in either CKD G3 or G4, indicating the potential utility of tolvaptan in patients older than the 18-55 year-olds who have been the focus of treatment recommendations to date.^5^ Tolvaptan initiation is known to induce a hemodynamic effect that results in an acute suppression of eGFR and is reversible with tolvaptan discontinuation, which is a potential concern for patients who already have low eGFR.^11^^,^^12^ However, an analysis of data on patients with very low baseline eGFR (15-29 mL/min/1.73 m^2^) who participated in REPRISE and the long-term extension indicated that tolvaptan significantly delayed further eGFR decline in this population over the follow-up period.^15^ The decision of patients with low baseline kidney function whether to initiate tolvaptan should be individualized in discussions with health care providers. Among all patients considering tolvaptan, possible treatment benefits must be weighed against known adverse effects, including drug-induced liver injury and aquaretic symptoms. Liver enzyme monitoring is required at treatment weeks 2 and 4, then monthly for the first 18 months of treatment. In general, aquaresis is greater in younger patients with preserved kidney function.^5^

Supporting the validity of the analyses, the rate of eGFR decline for the SOC group in the overall and matched populations (3-4 mL/min/1.73 m^2^ per year) was of a similar magnitude to the rates reported in the literature for ADPKD patients with substantial CKD progression at baseline and receiving non–disease-specific SOC. Annual eGFR decline rates were −3.9 mL/min/1.73 m^2^ with antihypertensive treatment in the HALT-PKD study B (required baseline eGFR 25-60 mL/min/1.73 m^2^)^13^; −3.5 mL/min/1.73 m^2^ in the Developing Intervention Strategies to Halt Progression of Autosomal Dominant Polycystic Kidney Disease (DIPAK) observational cohort study (required baseline eGFR 30-60 mL/min/1.73 m^2^), including in a subset of older patients receiving thiazide diuretics^20^; and −4.4 mL/min/1.73 m^2^ in study B of the MDRD trial (required baseline eGFR of 13-24 mL/min/1.73 m^2^).^21^ Even among ADPKD patients aged 50-60 years who were relatively low risk (Mayo Imaging Classes 1A-1B), the annual slope of eGFR decline was 3-4 mL/min/1.73 m^2^.^19^ The relatively lower eGFR annual decline in participants aged >55 years in the placebo arm of REPRISE (−2.34 mL/min/1.73 m^2^) compared with other cohorts (−3.5 to −4.4 mL/min/1.73 m^2^)^13^^,^^20^^,^^21^ may be explained by the fact that the patients in REPRISE randomized to placebo were slow progressors. In the present study, the eGFR annual decline was −3.99 mL/min/1.73 m^2^ in the SOC group matched by age, sex, and eGFR at baseline, which is comparable to other cohorts.

As with all nonrandomized studies using a control cohort for comparison, this analysis is limited by differences in study populations that may affect the outcomes. The use of multiple regression adjustment and participant matching reduced the potential for bias, but the possibility still exists.^9^ Data were lacking to enable matching by risk of rapid progression, for example by Mayo Imaging Class, with matching criteria limited to baseline age, sex, and kidney function. Evidence of rapid progression was defined in REPRISE as baseline eGFR 25-44 mL/min/1.73 m^2^ and historical decline >2 mL/min/1.73 m^2^ per year, but this definition was not used for inclusion in the present analysis. Historical data on eGFR decline were unavailable for the analysis, whereas such data would often be available in the clinic. Given that in older patients with ADPKD, aging-related factors such as vascular disease appear to be more important to the progression of CKD than cystic growth,^22^ the inclusion of vascular disease (eg, history of hypertension, dyslipidemia, or macrovascular disease) in the matching criteria would also have produced better matching. Such data was not systematically collected in a uniform way across studies, however, and may not have been complete. Another limitation is that the natural history of ADPKD precludes evaluating treatment effects on clinical endpoints such as mortality or the need for kidney replacement therapy within the time frame of a clinical trial. Decline in eGFR is, however, an acceptable intermediate endpoint in ADPKD for regulatory agencies, enabling clinical trial follow-up times of 2 years or less to assess pharmacotherapy.^4^^,^^23^^,^^24^

Despite these caveats, the large database used for this analysis enabled long-term comparison of kidney function trajectory between patients aged 56-65 years with advanced CKD and rapidly progressing disease who received tolvaptan treatment and a similar population who did not receive tolvaptan. The analysis did not include older patients with largely preserved eGFR and/or those progressing more slowly, and the findings accordingly do not provide information regarding the treatment of such patients. Those older than 55 and in worse ADPKD risk categories (eg, Mayo Imaging Class 1C through 1E) are predicted to reach kidney failure relatively rapidly, underscoring the need for effective intervention.^19^^,^^25^ In conclusion, the results from this pooled data study with a well-matched control cohort support the benefits of initiating treatment in an older population with advanced ADPKD (CKD G3 or G4) and evidence of rapid progression (ie, eGFR decline ≥3 mL/min/1.73 m^2^ per year).
