# Supplementary material for: Tolvaptan and Kidney Function Decline in Older Individuals With Autosomal Dominant Polycystic Kidney Disease: A Pooled Analysis of Randomized Clinical Trials and Observational Studies
Source: Kidney Med. 2023 Apr 14;5(6):100639. doi: 10.1016/j.xkme.2023.100639 (PMC10220412; doi:10.1016/j.xkme.2023.100639)
Supplement: Supplementary File (PDF) — Fig S1, Table S1-S3 [file mmc1.docx]

**Figure S1.** eGFR over time for each participant in the matched population


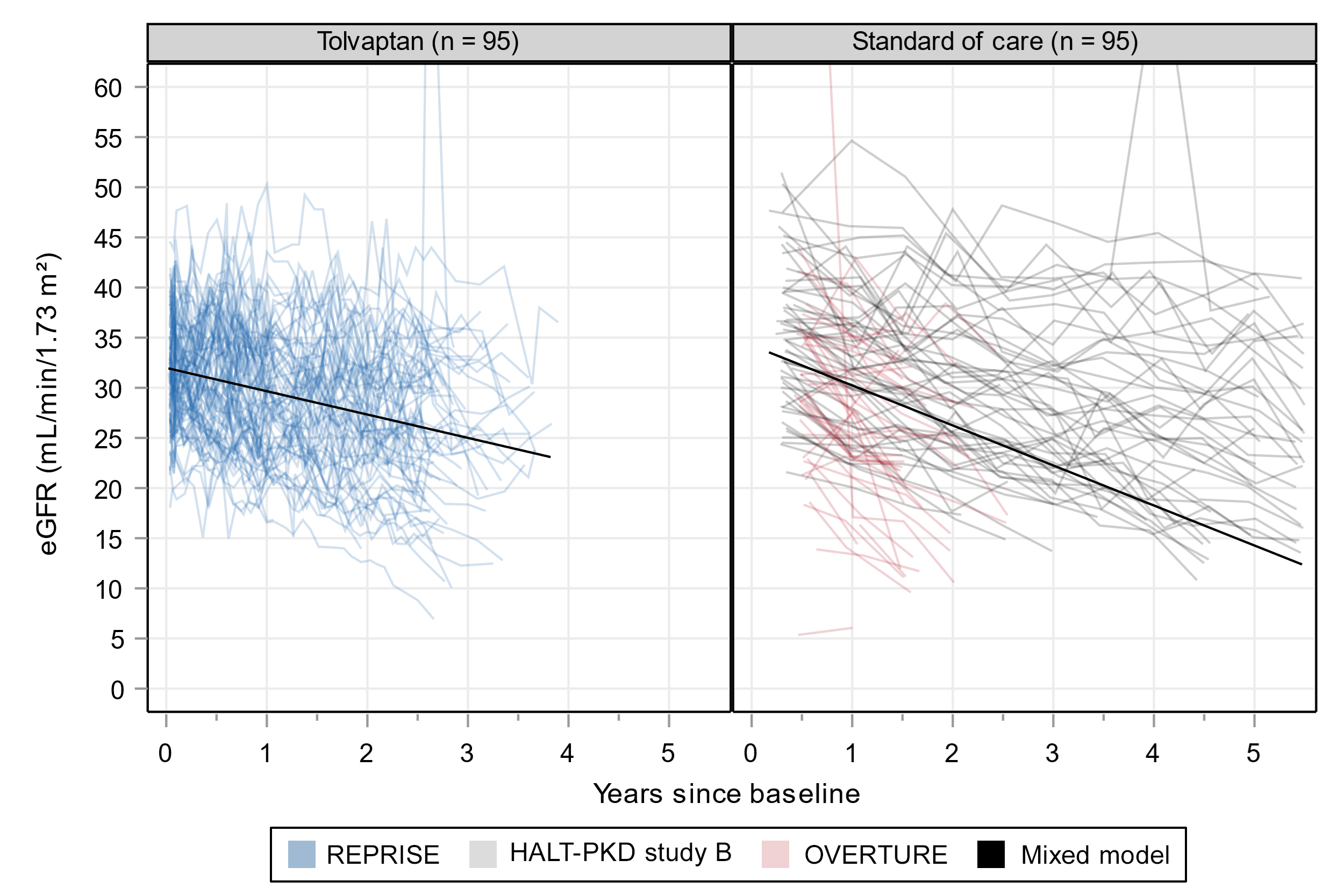


Three outlier data points (eGFR >60 mL/min/1.73 m^2^) are not displayed in the figure.

eGFR, estimated glomerular filtration rate.

**Table S1.** Studies included in the pooled database of participants aged >55 years

| **Study (NCT Number)** | **Description (Study Period)** | **Population Key Inclusion Criteria^a^** |
| --- | --- | --- |
| TEMPO 2:4 (NCT00413777) | Phase 2, TOL for 3 years plus 1-year extension  (Dec 2005-Jun 2010) | - Previous participation in designated TOL ADPKD phase 1 studies - ≥18 years of age - eGFR ≥30 mL/min/1.73 m^2^ by standard formula |
| 156-06-260  (No NCT number) | Phase 1, short-term study in participants  with different levels of kidney function  (Mar 2007-Feb 2010) | - No previous exposure to TOL - 18-60 years of age - 3 eGFR (Cockcroft-Gault) groups:  ≥60 mL/min/1.73 m^2^, 45 to <60 mL/min/1.73 m^2^, 30 to <45 mL/min/1.73 m^2^   BMI: 19-32 kg/m^2^ |
| 156-09-284  (NCT01336972) | Phase 2, short-term study in participants  with different levels of kidney function  (Oct 2010-Nov 2011) | - No previous TOL exposure - 18-70 years of age - 3 eGFR (MDRD) groups: >60 mL/min/1.73 m^2^, 30-60 mL/min/1.73 m^2^, <30 mL/min/1.73 m^2^ - BMI: ≤35 kg/m^2^ |
| TEMPO 4:4 (NCT01214421) | Extension trial (up to 5 years) of participants completing previous clinical trials  (May 2010-Feb 2016) | - Completion of a TOL ADPKD trial with or without previous TOL exposure - eGFR (MDRD) ≥30 mL/min/1.73 m^2^ (<30 mL/min/1.73 m^2^ were allowed per medical monitor approval) |
| REPRISE  (NCT02160145) | Phase 3b, randomized-withdrawal, PBO-controlled study with 1 year of treatment. Participants who tolerated at least 60/30 mg TOL in the 2-week TOL-titration period and completed the 3-week run-in period were randomized  (May 2014-Apr 2017) | - TOL naïve - 18-55 years of age with eGFR 25-65 mL/min/1.73 m^2^, or - 56-65 years of age with eGFR 25-44 mL/min/1.73 m^2^ with evidence of rapid ADPKD progression |
| 156-13-211  Long-term safety (NCT02251275) | Phase 3b, open-label 18-month extension trial for participants previously enrolled in other TOL trials  (Oct 2014-Nov 2018) | - ≥18 years of age - Completed REPRISE, TEMPO 4:4, or a prior ADPKD TOL trial; - or interrupted or discontinued TOL treatment in a prior trial other than REPRISE with medical monitor approval - Estimated eGFR ≥20 mL/min/1.73 m^2^ within 3 months of the baseline visit or with medical monitor and sponsor approval |
| OVERTURE  (NCT01430494) | Observational study (up to 3 years), TOL-naïve participants, as well as participants who discontinued clinical trials and agreed to further observation  (Jun 2011-Oct 2014) | - 12-70 years of age - TKV ≥300 cc/m height by ultrasound or ≥250 cc/m height by MRI - Enriched in participants likely to benefit from therapy (16-48 years, eGFR ≥45 mL/min/1.73 m^2^, TKV ≥400 cc/m height) |
| HALT-PKD Study B (NCT01885559) | Randomized study with a follow-up of 5-8 years (2006-2014)  ACEi + ARB with standard BP control^b^  ACEi with standard BP control^b^ | - Hypertension or high-normal BP - Ineligible if participants have kidney disease other than ADPKD or if they have diabetes - 18-64 years of age - Moderately advanced PKD defined by GFR (MDRD) 25-60 mL/min/1.73 m^2^ |

^a^ All studies required diagnosis of ADPKD. ^b^ Standard BP control is 110-130/70-80 mmHg.

ACEi, angiotensin-converting enzyme inhibitor; ARB, angiotensin receptor blocker; BMI, body mass index; BP, blood pressure; eGFR, estimated glomerular filtration rate; GFR, glomerular filtration rate; MDRD, Modification of Diet in Renal Disease; NCT number, clinicaltrials.gov identifier; PBO, placebo; PKD, polycystic kidney disease; TOL, tolvaptan; TKV, total kidney volume.

**Table S2.** Assessment schedules for eGFR in the source studies

| **Study** | **Assessment Schedule** |
| --- | --- |
| TEMPO 2:4 | Screening, day 1; weeks 1, 2, 3, 4; months 2, 6, 9, 12, 16, 20, 24, 28, 32, 36  Extension day 1, extension month 12, early termination |
| 156-06-260 | Baseline, day 8 |
| 156-09-284 (NCT01336972) | Screening, baseline; days 7, 14, 21; early termination; 3 weeks posttreatment |
| TEMPO 4:4 | Screening, baseline; months 1, 3, 6, every 6 months thereafter; early termination; follow-up (+7 days) |
| REPRISE | Screening, end of placebo run-in period (day -36), weeks 2, 4, and 5 of tolvaptan titration/run-in period (days -22, -8, and -1)  Months 1 to 12 of the randomized treatment period, end of treatment, 3 visits in between 8 to 21 days after the last dose |
| 156-13-211  Long-term safety | Screening, monthly, early termination/end of treatment, 7 days posttreatment |
| OVERTURE | Baseline, months 6, 12, 18, every 6 months thereafter; early termination |
| HALT-PKD Study B | Screening, baseline, week 16, months 12, 18, and every 6 months thereafter |

eGFR, estimated glomerular filtration rate.

**Table S3.** Tolvaptan treatment duration, compliance, and gap between studies (all participants aged >55 years)

|  |  |  | **Initial Study (Followed by Extension Study)** | | |
| --- | --- | --- | --- | --- | --- |
| **Variable** | **Statistic or Category** | **Tolvaptan**  **(n=230)** | **REPRISE  Tolvaptan Arm ^a^**  **(n=95)** | **REPRISE Other^b^**  **(n=118)** | **Other Studies**  **(n=17)** |
| Treatment duration, years | n | 230 | 95 | 118 | 17 |
|  | Mean (SD) | 2.25 (1.49) | 2.39 (1.02) | 1.89 (1.36) | 3.89 (2.85) |
|  | Median | 2.66 | 2.69 | 2.54 | 3.76 |
|  | Min, Max | 0.00, 9.18 | 0.18, 3.90 | 0.00, 3.94 | 0.02, 9.18 |
|  | ≥3 years | 65 (28.3%) | 27 (28.4%) | 28 (23.7%) | 10 (58.8%) |
|  | ≥5 years | 5 (2.2%) | 0 (0.0%) | 0 (0.0%) | 5 (29.4%) |
| On treatment time, years | n | 230 | 95 | 118 | 17 |
|  | Mean (SD) | 1.78 (1.33) | 2.30 (1.01) | 1.15 (0.94) | 3.29 (2.41) |
|  | Median | 1.84 | 2.58 | 1.47 | 3.20 |
|  | Min, Max | 0.00, 8.38 | 0.18, 3.83 | 0.00, 2.88 | 0.02, 8.38 |
|  | ≥3 years | 34 (14.8%) | 24 (25.3%) | 0 (0.0%) | 10 (58.8%) |
|  | ≥5 years | 2 (0.9%) | 0 (0.0%) | 0 (0.0%) | 2 (11.8%) |
| Treatment compliance, % | n | 230 | 95 | 118 | 17 |
|  | Mean (SD) | 81.7% (22.0%) | 95.6% (7.1%) | 69.4% (23.8%) | 88.3% (12.6%) |
|  | Median | 95.7% | 97.8% | 66.1% | 92.8% |
|  | Min, Max | 12.6%, 100.0% | 56.7%, 100.0% | 12.6%, 100.0% | 64.6%, 100.0% |
|  | ≥70% | 156 (67.8%) | 93 (97.9%) | 48 (40.7%) | 15 (88.2%) |
| Received tolvaptan in ≥2 studies/study extension | n (%) | 167 (72.6%) | 73 (76.8%) | 81 (68.6%) | 13 (76.5%) |
| Maximum gap duration between studies/study extension, years | n | 167 | 73 | 81 | 13 |
|  | Mean (SD) | 0.60 (0.53) | 0.08 (0.06) | 1.06 (0.06) | 0.68 (0.84) |
|  | Median | 1.00 | 0.06 | 1.04 | 0.31 |
|  | Min, Max | 0.03, 2.35 | 0.03, 0.46 | 1.00, 1.48 | 0.08, 2.35 |
| Total gap duration between studies/study extension, years | n | 167 | 73 | 81 | 13 |
|  | Mean (SD) | 0.60 (0.53) | 0.08 (0.06) | 1.06 (0.06) | 0.72 (0.84) |
|  | Median | 1.00 | 0.06 | 1.04 | 0.31 |
|  | Min, Max | 0.03, 2.35 | 0.03, 0.46 | 1.00, 1.48 | 0.08, 2.35 |
| Gap duration prior to TEMPO 2:4 extension, years | n | 2 | n/a | n/a | 2 |
|  | Mean (SD) | 0.31 (0.00) |  |  | 0.31 (0.00) |
|  | Median | 0.31 |  |  | 0.31 |
|  | Min, Max | 0.30, 0.31 |  |  | 0.30, 0.31 |
| Gap duration prior to TEMPO 4:4, years | n | 13 | n/a | n/a | 13 |
|  | Mean (SD) | 0.68 (0.84) |  |  | 0.68 (0.84) |
|  | Median | 0.31 |  |  | 0.31 |
|  | Min, Max | 0.08, 2.35 |  |  | 0.08, 2.35 |
| Gap duration prior to long-term safety extension, years | n | 154 | 73 | 81 | n/a |
|  | Mean (SD) | 0.59 (0.50) | 0.08 (0.06) | 1.06 (0.06) |  |
|  | Median | 1.01 | 0.06 | 1.04 |  |
|  | Min, Max | 0.03, 1.48 | 0.03, 0.46 | 1.00, 1.48 |  |
| Prior exposure to tolvaptan | n | 230 | 95 | 118 | 17 |
|  | Yes | 3 (1.3%) | 0 (0.0%) | 0 (0.0%) | 3 (17.6%) |
|  | No | 227 (98.7%) | 95 (100.0%) | 118 (100.0%) | 14 (82.4%) |

^a^ The REPRISE investigators reported 96 participants aged >55 years who were randomized to tolvaptan (Torres VE et al. *N Engl J Med.* 2017;377:1930-42). The pooled analysis counted participants by age at baseline, not randomization, resulting in a population of 95 participants. ^b^ Participants in REPRISE who received tolvaptan in the pre-randomization titration/run-in period and who were then not randomized to the tolvaptan study arm. Most were randomized to the placebo arm, but a few did not enter the randomized phase.

Max, maximum; min, minimum; SD, standard deviation.
